# A protocol for the gram-scale synthesis of polyfluoroaryl sulfides via an S_*N*_Ar step

**DOI:** 10.1016/j.xpro.2023.102043

**Published:** 2023-01-19

**Authors:** Cheng-Lin Chan, Shao-Chi Lee, Hsuan-Hung Liao

**Affiliations:** 1 Department of Chemistry, National Sun Yat-sen University, Kaohsiung 80424, Taiwan; 2 KAUST Catalysis Center (KCC), King Abdullah University of Science and Technology (KAUST), Thuwal 23955-6900, Saudi Arabia

**Keywords:** NMR, Chemistry, Material Sciences

## Abstract

Polyfluoroaryl sulfide is one of the prevalent motifs ubiquitous in materials and pharmaceutical chemistry. We herein describe a simple yet efficient procedure for their synthesis from readily available thiols and polyfluoroarenes via an S_*N*_Ar step. We detail specific steps for a gram-scale preparation of 2-((perfluoropyridin-4-yl)thio)benzo[*d*]thiazole **3** from mercaptobenzothiazole **1** and pentafluoropyridine **2**.

For complete details on the use and execution of this protocol, please refer to Liao et al. (2022).^1^

## Highlights

• A general approach for sulfurization of perfluoroarenes at gram scale
• High regioselectivity and functional group compatibility
• The protocol is limited to aryl fluorides

## Abstract (editor highlights)

Publisher’s note: Undertaking any experimental protocol requires adherence to local institutional guidelines for laboratory safety and ethics.

## Graphical abstract

**Figure:**
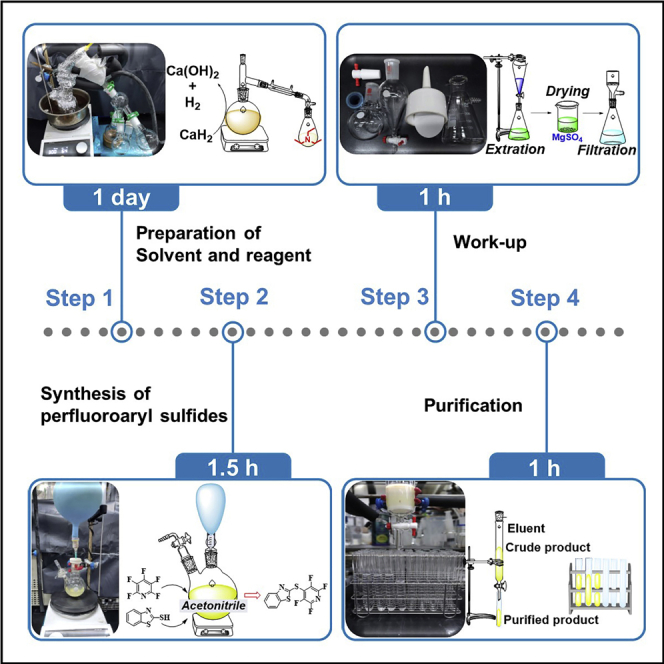

## Before you begin

Aromatic thioethers are attractive targets for the development of new efficient synthesis protocols being a recurring motif in bioactive compounds,^2^^,^^3^ pharmaceuticals,^4^^,^^5^ and materials science.^6^^,^^7^ This predilection is further amplified with the limitations purported from reported literature methods such as poor regioselectivity and the necessity of transition metals.^8^^,^^9^ Other protocols for the synthesis of polyfluoroaryl sulfides are also rather complex; requiring hetarenium salts that act as an activated substrate for thiolation^10^^,^^11^ or are comported into focused settings such as in the preparation of polymers^12^ and in peptide stapling.^13^^,^^14^

We further reckoned that a straightforward, metal-free approach for the synthesis of polyfluoroaryl sulfides could provide a solution to the previously mentioned challenges. With S_*N*_Ar as a viable pathway, a general protocol that is regioselective, cheap, and simple is disclosed to provide *para*-thiolated polyfluoroarenes in a wider substrate scope, lower overall cost, and good to excellent yields. Additionally, this protocol is amenable to late-stage functionalization of natural product derivatives and is commutable to solvent-free mechanical ball milling^15^^,^^16^ and flow reaction,^17^ please refer to Liao et al.^1^ We have also previously applied this protocol for the installation of pentafluoropyridines in benzyl mercaptans and mercaptoacetates, which activates the latter two for the desulfurative nickel-catalyzed reductive Liebeskind−Srogl type cross-coupling with aryl halides.^18^

### Preparation of the reagents and equipment

A complete list of reagents and equipment can be found in the “key resources table”.

#### Purification of reagent – Triethylamine

**Timing: 1 day**

In this step, the purification of triethylamine is described. Triethylamine stored for a long time may contain excessive impurities and/or moisture. This step can be skipped if using brand new bottle of triethylamine. According to our test, the quality of triethylamine will affect the yield by about 10%–15% in this substrate.

**Figure 1:**
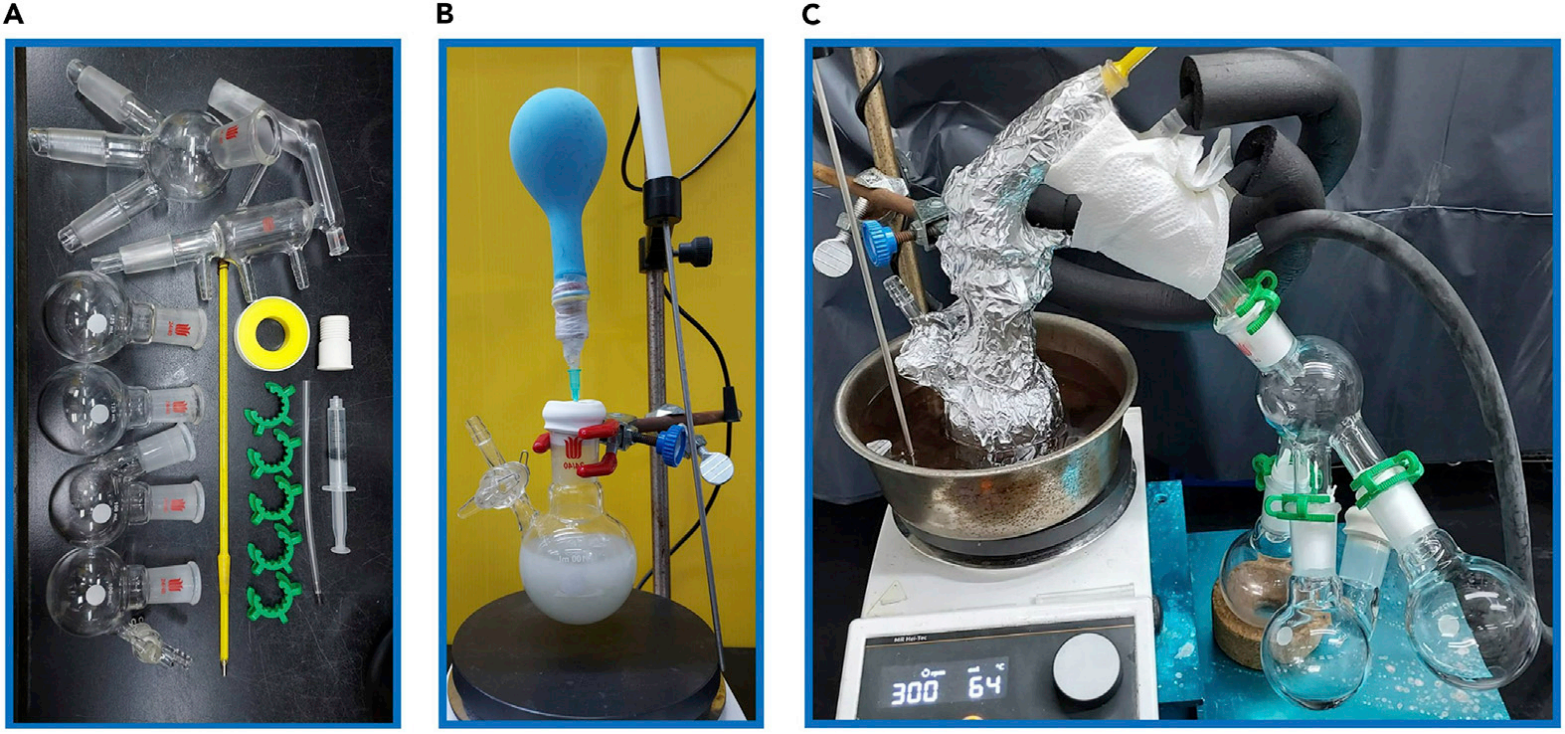
Overview of the purification system (A) Required accessories. (B) Schematic of processing. (C) Set up of distillation.

1. Preprocessing.
  a. Carefully weigh 1084.4 mg CaH_2_ in a 100 mL oven-dried single-neck round bottom flask equipped with 6 mm∗ 24 mm teflon stir-bar.
  b. Add 60 mL of triethylamine. Troubleshooting 1.
  c. Seal the round bottom flask with a rubber septum and lock the system by inserting a nitrogen balloon through an inlet needle.
  d. Stir the mixture at room temperature overnight (14 h) (Figure 1B).

2. Distillation.
  a. Set-up the distillation apparatus and connect the condenser to the source of cold water, keep the distillation system under nitrogen through the Schlenk line.
  b. Turn on the condenser. Confirm that there is no water leakage.
  c. Connect the CaH_2_-pretreated triethylamine flask to the distillation apparatus and fix with a joint clip.
  d. Wrap the condenser with cloth or tissue paper to prevent the condensed water from dripping into the oil bath (Figure 1C).
    ***Alternatives:*** Oil bath can be replaced with heating mantle or aluminum heating blocks.
  e. Cover the distilling flask with aluminum paper to maintain uniform heating (Figure 1C).
  f. Slowly increase the temperature to 80°C and collect the triethylamine distillate in three (3) separate round-bottom flasks connected through a cow-type receiver.
    **CRITICAL:** Do not distill all the triethylamine. Keep about 10–15 mL to avoid any danger from the residual CaH_2_. The residual CaH_2_ should be gradually quenched with alcohols (such as methanol) to ice water in an ice bath.
  g. Use H-NMR to identify whether it contains impurities. Troubleshooting 2.

## Key resources table

**Table:** 

REAGENT or RESOURCE	SOURCE	IDENTIFIER
Chemicals, Peptides, and Recombinant Proteins		

HPLC-grade acetonitrile	J.T. Baker	CAS: 75-05-8
Gradient-grade acetonitrile	Merck	CAS: 75-05-8
ACS ethyl acetate	Macron	CAS: 141-78-6
ACS hexane	Duksan	CAS: 110-54-3
Calcium hydride	Acros	CAS: 7789-78-8
2-Mercaptobenzothiazole	Sigma-Aldrich	CAS: 149-30-4
Molecular sieve	Alfa Aesar	CAS: 308080-99-1
Pentafluoropyridine	BLDpharm	CAS: 700-16-3
Silica gel for chromatography	KM3	CAS: 7631-86-9
Ammonium chloride	VETEC	CAS: 12125-02-9
Triethylamine	Alfa Aesar	CAS: 121-44-8

Other		

Electronic balance	Shimadzu	UW2200H/ATX224
Magnetic stirrer	Corning	PC-420D
Pump of rotary evaporator	KNF Laboport	N820.3FT.18
Refrigerated circulator bath	Panchum	CC-1000/CC-2000/CC-3000
Rotary evaporator	Heidolph	Hei-Vap Core HL G3
Vacuum pump	Edwards	RV5

## Step-by-step method details

### Synthesis of sulfide

**Timing: 1.5 h**

**Scheme 1:**
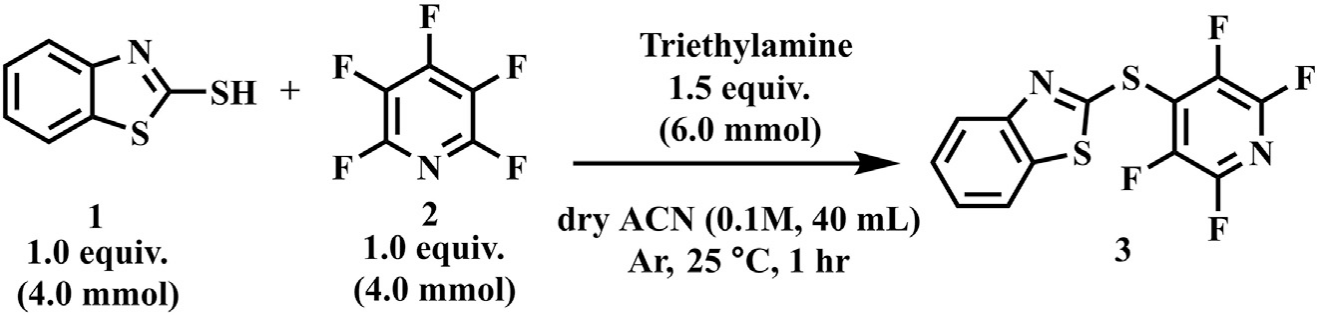
General scheme of the reaction

In this step, the detailed synthesis procedure of 2-((perfluoropyridin-4-yl)thio)benzo[*d*]thiazole **3** has been described (Scheme 1).

**Figure 2:**
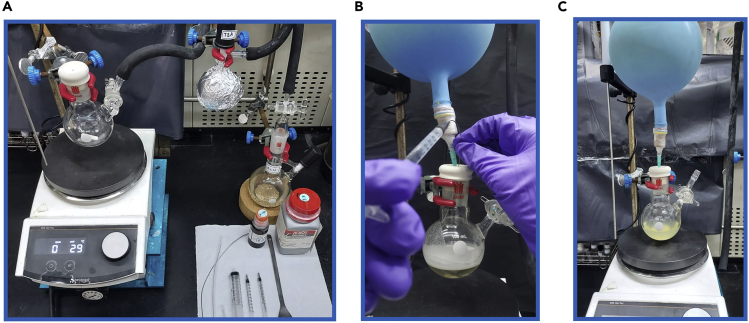
Overview of the reaction set up (A) Required accessories. (B) Inject reagent. (C) Schematic of reaction processing.

1. Reaction setting up.
  a. Connecting the distilled triethylamine storage bottle from step 1 (before you begin) to the Schlenk line and keep the bottle in a positive pressure with nitrogen.
  b. Use a spatula to weigh 670.5 mg (4.0 mmol) of 2-mercaptobenzothiazole **1** on the folded weighing paper in the zeroed balance. Troubleshooting 3.
  c. Connect a two-neck round-bottomed flask to a Schlenk line and under nitrogen flow load the 2-mercaptobenzothiazole **1.** Subsequently, seal the flask with a rubber septum. Purge with nitrogen for three times and keep for positive pressure before next step.
    ***Note:*** If the flow rate of nitrogen is too strong, the solids may be blown away. Adjust the pressure to an appropriate flow rate.
  d. Remove the reaction flask from the Schlenk line and fix it on the stirring plate with three prong flask clips and nitrogen balloon.
  e. The anhydrous acetonitrile solvent storage bottle was connected to the Schlenk line. Under nitrogen blowing, utilize 50 mL syringe to transfer 40 mL of anhydrous acetonitrile into the reaction bottle.
    ***Note:*** Although reagent-grade acetonitrile gave similar results in the yield of this reaction (from 85% to 83%) compared to anhydrous HPLC-grade acetonitrile, considering the suitability for different substrates, the authors recommend the use of higher purity solvent with appropriate treatment (e.g., adding molecular sieves, using freeze-pump-thaw technique or sparging with nitrogen/argon to remove water and/or air) and storing it under an inert atmosphere.
  f. Add 0.836 mL of distilled triethylamine into the reaction flask with a 1 mL plastic syringe.
  g. Inject 0.439 mL (4.0 mmol) pentafluoropyridine into the reaction flask with a 1 mL plastic syringe (Figure 2B).
  h. Power on the stirring plate and set the stirring speed to 600 rpm (Figure 2C).
    ***Note:*** Pentafluoropyridine has a low boiling point, seal reaction flask to avoid spillage.

### Tracking reactions and purification

**Timing: 2 h**

This step describes how to determine if the reaction is completed, and how to obtain the final product **3** by purification.

**Figure 3:**
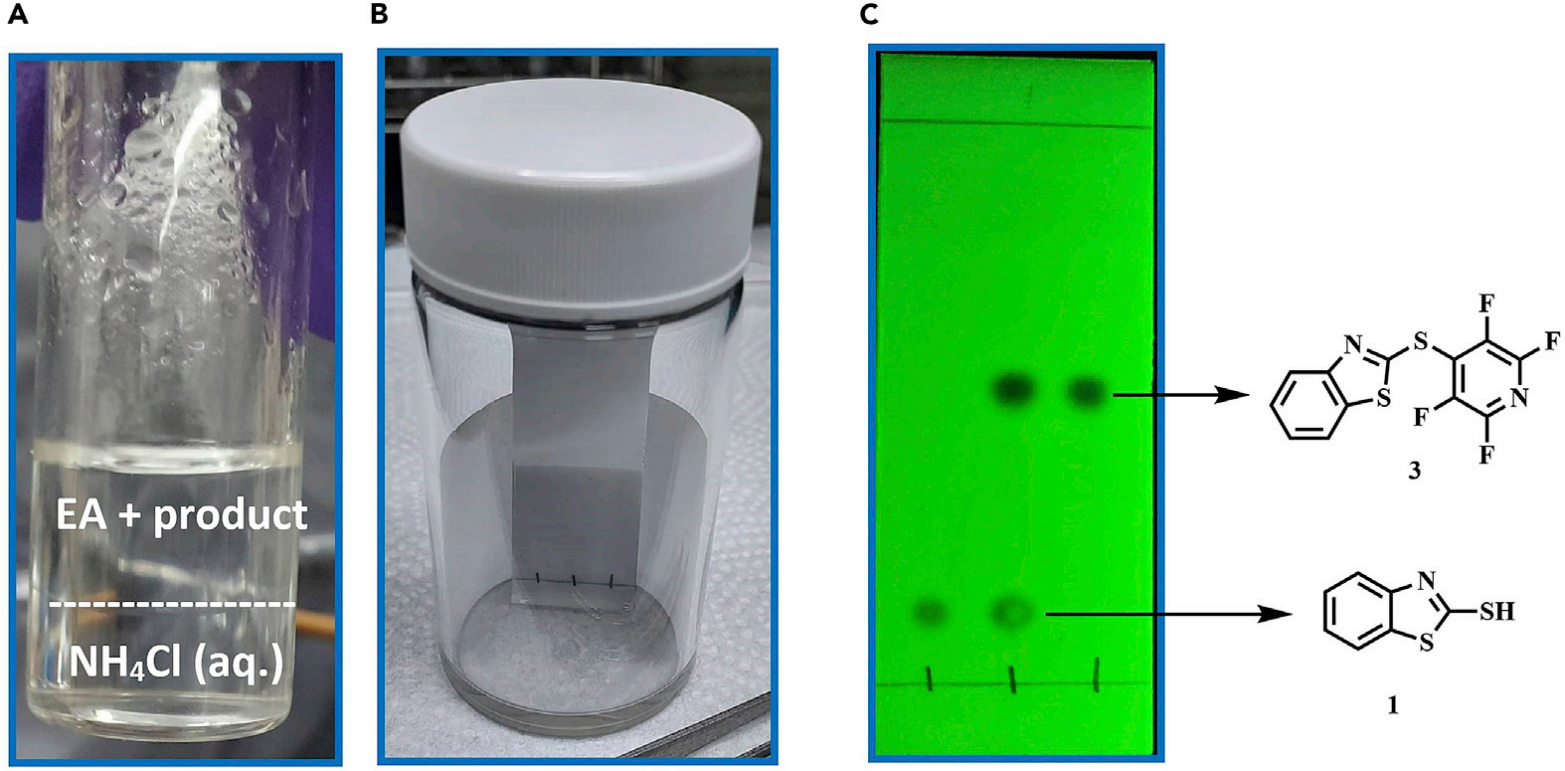
Overview of reaction tracking (A) The mixture of organic/aqueous layer. The boundary line is highlighted as which dash line. (B) TLC plate in the camber. (C) Fluorescent TLC plate under an UV-light (254 nm). The upper spot is the target product **3**.
  b. Use a capillary to dip the organic layer solution (generally, EtOAc will be in the upper layer), spot on thin-layer chromatography plate with the perfluoroarene starting material and thiol.
  c. Use hexane as the mobile phase and place the TLC plate in the sealed camber (Figure 3B). Take the TLC plate out when the R_f_ value is around 0.8.
  d. Confirm whether the reaction is complete by 254 nm UV lamp (Figure 3C). If perfluoroarene starting material remains, extend the reaction time until it is well-consumed.

2. Tracking reactions.
  a. Microscale extraction - After the reaction time is up, use a 1 mL syringe to draw 0.2 mL of the reaction solution into 4 mL vial which contains 1 mL of EtOAc and 1 mL of saturated NH_4_Cl_(aq)_. Close the lid and shake it about 20 times, then stand for 30 s before opening the lid (Figure 3A). Troubleshooting 4.

**Figure 4:**
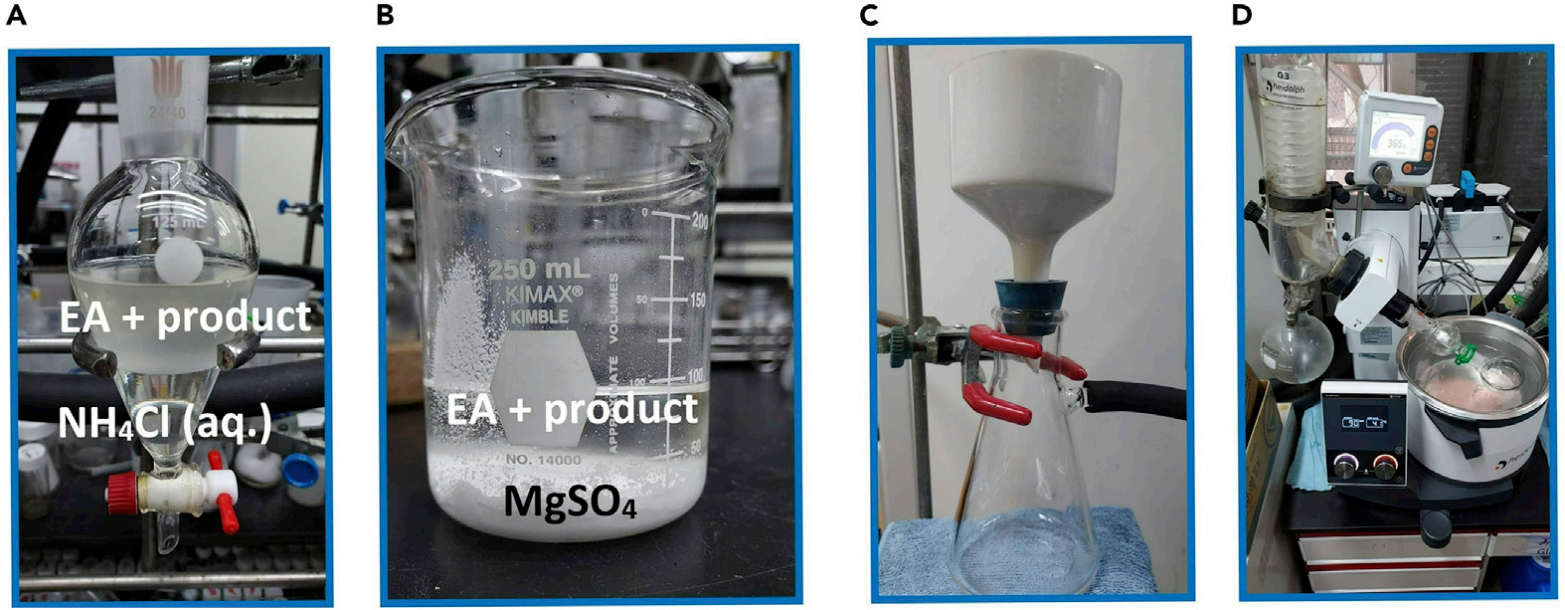
Overview of reaction work-up (A) Layering in a separatory funnel. The upper layer is the organic/product layer. (B) Remove water from collected organic by anhydrous magnesium sulfate. (C) Filter anhydrous magnesium sulfate with Buchner funnel. (D) Utilizing rotary evaporator to remove the solvent from filtrate.

**Figure 5:**
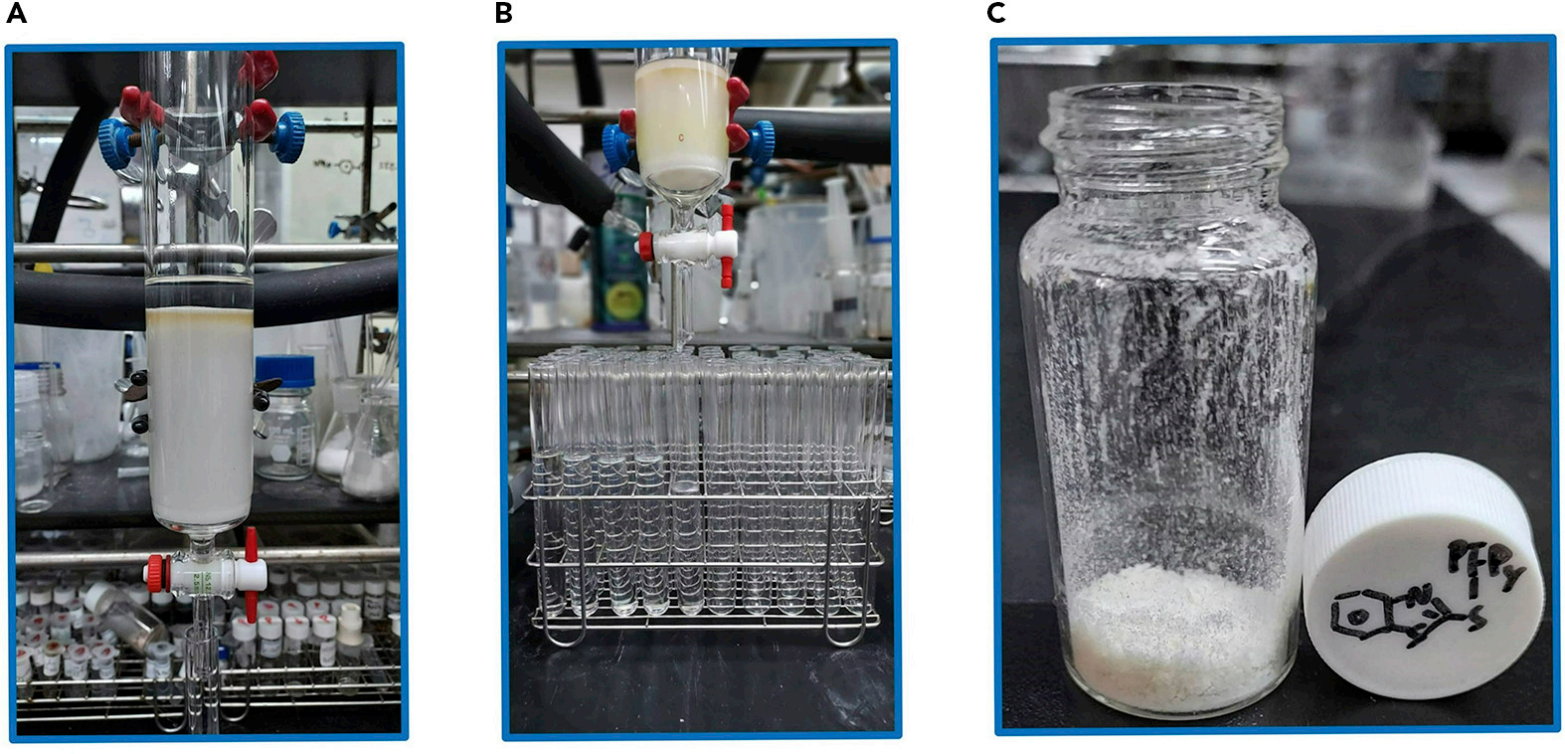
Overview of column chromatography (A) Packing the column. (B) Collect the effluent solution in a test tube. (C) Final product 2-((perfluoropyridin-4-yl)thio)benzo[*d*]thiazole **3**.

3. Extraction.
  a. After confirming the termination of the reaction (it takes 1 h in this example reaction), rinse the vial with 20 mL EtOAc and transfer the reaction mixture into a 125 mL separatory funnel.
  b. Add 20 mL of saturated NH_4_Cl_(aq)_ into the separatory funnel and close it with a Teflon stopper. Shaking the funnel until layering is separated (Figure 4A). Troubleshooting 4.
  c. The aqueous layer was collected in a 100 mL beaker, then repeating the step 3b for three times. The well-extracted organic layer was placed in a 100 mL beaker.
  d. Pour the beaker containing the aqueous layer into a separatory funnel, extract with 20 mL EtOAc and combine the organic layers in the same beaker.
  e. Add 2 g of anhydrous magnesium sulfate to the beaker containing the organic layer, stir well with a glass rod until no suspended particles float in the solution (Figure 4B).
    ***Note:*** If no suspension of anhydrous magnesium sulfate is observed, add 1 g at a time. Excessive addition may result in decreased yield.
  f. The magnesium sulfate was removed by suction filtration with 70 mm filter paper and Büchner funnel, further rinsed twice with 15 mL EtOAc (Figure 4C).
    ***Alternatives:*** Filter paper and Büchner funnel can be replaced by glass funnel filter.
  g. The filtrate was collected in a 125 mL round bottom flask, and the solvent was removed by a rotary evaporator under 180 mbar and 40°C for 30 min (Figure 4D).

4. Purification Troubleshooting 5.
  a. Dissolve the reaction mixture with 30 mL dichloromethane and 2 g of silica gel.
  b. Remove the solvent with a rotary evaporator under 750 mbar and 40°C for 15 min to obtain the mixture attached to the silica gel.
    ***Note:*** Silica gel is easy to erupt during evaporation, cotton can be stuffed into bump trap (anti-splash adapter) to prevent rotary evaporator damage.
  c. Dissolve 42 g of silica gel with mixture solvent (100 mL of Hexane and 10 mL Ethyl acetate), add into the column and backfill the extruded solvent into the column for 5 times under the pressure until the silica gel is tightly packed.
  d. Use plastic funnel to slowly add the silica gel of step 4b (Figure 5A).
    ***Note:*** Retaining about 0.5 cm of solvent to prevent impact on the surface when pouring the silica gel.
  e. Pave a layer of sea sand on the top, then begin the column chromatography with the hexane and ethyl acetate (ratio 10:1) as eluent.
  f. Collet the sample with 11 mL test tube. Confirm presence of 2-((perfluoropyridin-4-yl)thio)benzo[*d*]thiazole **3**, as in steps 2b–2d (step-by-step method details) (Figure 5B).
  g. Pour the test tubes in which the desired product was detected into a 250 mL round bottom flask, remove the solvent with a rotary evaporator under 300 mbar and 40°C for 30 min.
  h. Further remove the remaining solvent by using a high vacuum for 15 min.
  i. The final product was obtained as white solid (1084.0 mg, 0.3428 mmol, 85.7% isolated yield) (Figure 5C). Troubleshooting 6.

## Expected outcomes

2-((perfluoropyridin-4-yl)thio)benzo[*d*]thiazole **3** appears as a white solid obtained in 85% yield.

### Analytical data

^**1**^**H NMR** (300 MHz, CDCl_3_) *δ* 7.93–7.90 (m, 1H), 7.83–7.80 (m, 1H), 7.51–7.46 (m, 1H), 7.43–7.38 (m, 1H) ppm.

^**13**^**C{**^**1**^**H} NMR** (101 MHz, CDCl_3_) *δ* 157.7, 152.7, 136.2, 126.8, 125.8, 122.9, 121.3 ppm.

^**19**^**F NMR** (376 MHz, CDCl_3_) *δ* -88.4 (s, 2F), -132.6 (s, 2F) ppm.

**IR** (ATR) ν_max_/cm^-1^: 2923, 2856, 1629, 1458, 1237, 991, 947 and 760 cm^-1^.

**HRMS** (EI) *m/z*: [M]^+^ Calcd. for C_12_H_4_F_4_N_2_S_2_ 315.9747; found 315.9749.

**M.p.**: 92°C.

**TLC**: R_*f*_ = 0.67 (Hexane/ethyl acetate 10:1).

## Limitations

The protocol is limited to aryl fluorides. Complete results on functional group compatibility can be found in our previously published article (Liao et al.^1^).

## Troubleshooting

### Problem 1

Step 1b (before you begin): Triethylamine has an unbearable stench.

### Potential solution

The boiling point of triethylamine is only 89°C (192°F), it is easy to volatilize at room temperature and produce an unpleasant odor. Therefore, all operations should be performed in a well-ventilated fume hood, and the used syringes or glassware should be cleaned immediately after use.

### Problem 2

Step 2g (before you begin): The H-NMR spectrum indicates that the distilled triethylamine still contains impurities.

### Potential solution

Carefully confirm the following precautions to ensure the purity of the distilled reagents.

• Heating the mixture gradually and stirring smoothly with a magnet stirrer to avoid bumping.
• Collecting the samples in sections with several flasks (Figure 1C).
• If the above steps have been strictly followed but there are still impurities, try the other fresh bottle of triethylamine.

### Problem 3

Step 1b (step-by-step method details): Mercaptan (Thiol) has an unbearable stench.

### Potential solution

All operations should be performed in a well-ventilated fume hood. If the balance is not in a fume hood, use a beaker instead of the weighing paper and cover it by a lid to reduce odor emissions. Glassware with mercaptan (thiol) residues can be cleaned by soaking in bleach.

### Problem 4

Step 2a and 3b (step-by-step method details): Solvent spills during shaking, and the layering is not obvious after shaking.

### Potential solution

Due to the volatility of organic solvents, part of the solvent will turn into gas after shaking. Try to shake the vial lightly for five times and open the cap to release the gas pressure in the vial.

If the layer does not separate well, add some brine can resolve the emulsion.

### Problem 5

Step 4 (step-by-step method details): After column chromatography the final product is still not pure.

### Potential solution

The following fine-tuning can improve the performance of column chromatography in separating products.

• Increase the amount of silica gel in the column during packing (step 4c, step-by-step method details).
• Reduce the proportion of polar solvent, which is ethyl acetate in this case in the mobile phase.

### Problem 6

Step 4i (step-by-step method details): Yield is lower than expected.

### Potential solution

This reaction has good reproducibility, if the yield is much lower than we report here, try to inspect the following notes.

• When filtering anhydrous magnesium sulfate, rinse several times with solvent to avoid product residue. Step 3f (step-by-step method details).
• In step 4d (step-by-step method details), if there is still residue in the bottle, rinse with eluent to ensure that all compounds have entered the column.

## Resource availability

### Lead contact

Further information and requests for resources and reagents should be directed to and will be fulfilled by the lead contact, Shao-Chi Lee (shaochi.lee@kaust.edu.sa).

### Materials availability

This study did not generate new unique reagents.

## Data Availability

• This study did not generate code. • Original data for substrate scope and the detail of ball milling/flow techniques please refer to our previous article (Liao et al.^1^). • Any additional information required to reanalyze the data reported in this paper is available from the lead contact upon request.
